# The Many Faces of MLKL, the Executor of Necroptosis

**DOI:** 10.3390/ijms241210108

**Published:** 2023-06-14

**Authors:** Veronica Martinez-Osorio, Yasmin Abdelwahab, Uris Ros

**Affiliations:** Institute for Genetics and Cologne Excellence Cluster on Cellular Stress Responses in Aging-Associated Diseases (CECAD), University of Cologne, 50931 Cologne, Germany; v.martinezosorio@smail.uni-koeln.de (V.M.-O.); yabdelwa@uni-koeln.de (Y.A.)

**Keywords:** MLKL, membrane permeabilization, necroptosis, regulated cell death, human diseases

## Abstract

Necroptosis is a recently discovered form of regulated cell death characterized by the disruption of plasma membrane integrity and the release of intracellular content. Mixed lineage kinase domain-like (MLKL) protein is the main player of this cell death pathway as it mediates the final step of plasma membrane permeabilization. Despite the significant progress in our knowledge of the necroptotic pathway and MLKL biology, the precise mechanism of how MLKL functions remain unclear. To understand in what way MLKL executes necroptosis, it is crucial to decipher how the molecular machinery of regulated cell death is activated in response to different stimuli or stressors. It is also indispensable to unveiling the structural elements of MLKL and the cellular players that are required for its regulation. In this review, we discuss the key steps that lead to MLKL activation, possible models that explain how it becomes the death executor in necroptosis, and its emerging alternative functions. We also summarize the current knowledge about the role of MLKL in human disease and provide an overview of existing strategies aimed at developing new inhibitors that target MLKL for necroptosis intervention.

## 1. Introduction

In contrast to accidental necrosis, apoptosis was long considered the only form of regulated cell death. However, the concept of regulated necrosis (RN) emerged to describe a type of cell death that phenotypically resembles necrosis, but, rather than accidental, it is triggered upon stimulation [1]. There are different forms of RN, including necroptosis, pyroptosis, and ferroptosis. They all converge in a final step of the plasma membrane disruption, the subsequent release of Damage-Associated Molecular Patterns (DAMPs), and the induction of inflammation and activation of immune responses [2]. Although different forms of RN and apoptosis have their own defined machinery, they all communicate and cross-regulate [3]. Therefore, the type of cell death predominating depends on the activation or deactivation of the molecular regulatory players involved in each pathway [4].

Necroptosis is a caspase-independent form of RN that relies on the activity of the pseudokinase Mixed Lineage Kinase domain-Like (MLKL) protein [5]. Upon activation by Receptor-Interacting Protein Kinase 3 (RIPK3), MLKL forms oligomers and translocates to the plasma membrane, causing cell death [6]. However, the molecular mechanism of how MLKL mediates the execution of necroptosis is still unclear. Likely due to its central role in the pathway, the membrane destabilizing activity of MLKL is tightly regulated in the cell and is connected with the physiological consequences of necroptosis [7]. In this regard, accumulating evidence in pre-clinical studies suggests that necroptosis is involved in a wide range of pathological conditions and diseases [8]. This poses MLKL as a potential target for interventions that aim to treat life-threatening diseases through the medicinal regulation of necroptosis. 

In this review, we provide a comprehensive overview of the molecular mechanism and the biological relevance of MLKL. We cover different aspects of MLKL biology, ranging from the activation of the necroptotic pathway and its relation to other types of cell death to the structural determinants and regulatory steps that determine its function as a death effector. Finally, we summarize the direct involvement of MLKL in the pathology of different human diseases and provide an overview of the current efforts to target MLKL with small molecules for research and clinical purposes.

## 2. The Necroptotic Pathway and Its Relation to Other Types of Regulated Cell Death

Necroptosis was first described as a type of necrotic cell death alternative to apoptosis occurring in mouse fibroblasts in response to the Tumor Necrosis Factor (TNF) [9]. Ten years later, the same type of cell death was associated with the activation of the Fas/CD95 signaling pathway activation in murine fibrosarcoma cells when caspases were inhibited [10]. Necroptotic cells differ morphologically from apoptotic cells in that the plasma membrane blooms, and the nucleus does not undergo disintegration [9]. It is now known that necroptosis occurs in response to various stimuli that implicate the activation of different receptors or sensing proteins. These include cell death receptors [11], pathogen-associated molecular pattern receptors [12], and intracellular RNA and DNA sensors [13]. These initial inputs lead to the subsequent formation of protein complexes that are essential to determine the outcome of the pathway. Indeed, such complexes are common hubs of pro-survival signaling and different types of cell death, including apoptosis, pyroptosis, and necroptosis [14]. Therefore, the fate of the cell greatly depends on the activation or repression of different regulatory players, such as caspases or the inhibitor of apoptosis proteins (IAPs) [15,16]. In this section, we will discuss the alternative signaling pathways that can lead to necroptosis and their relationship with other types of regulated cell death.

### 2.1. The Canonical TNF Pathway

**Figure 1:**
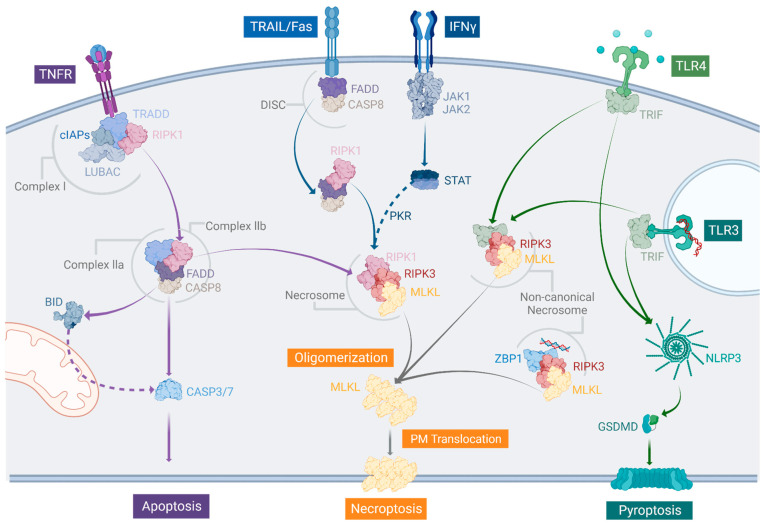
The multiple ways in which necroptosis can be induced and their relationship with other associated cell death pathways. Upon ligand binding, different receptors at the plasma membrane, such as TNFR, TRAIL, Fas/CD95, IFNγ, and TLR4 are activated, along with the subsequent signaling pathways. TNFR activation leads to the formation of complex I. When cIAPs are inhibited, RIPK1 is enabled to form complex IIb with FADD and caspase-8 (CASP8). Alternatively, complex IIa is formed by TRADD, FADD, and caspase-8. Both complexes IIa and IIb can activate caspase-8, which in turn induces apoptosis by cleaving different substrates, such as BID and the effector caspases 3 and 7 (CASP3/7). If caspase-8 is inhibited, then RIPK1 is recruited to form the necrosome with RIPK3 and MLKL for the induction of necroptosis. Stimulation of the TRAIL and Fas/CD95 pathways leads to DISC formation, RIPK1 recruitment, and the activation of caspase-8. As in the TNF pathway, caspase-8 can induce apoptosis, however, if it is inhibited, necrosome formation occurs. The IFN𝛾 pathway also activates RIPK1 by the JAK/STAT-mediated transcription of PKR, which interacts with RIPK1 for necrosome formation. The non-canonical necrosome can be formed via TRIF, after TLR3 and 4 activations, or when ZBP1 binds Z-DNA and Z-RNA intracellularly. TRIF can activate the NLRP3 inflammasome for the caspase-1-dependent cleavage of GSDMD to induce pyroptosis. Solid lines indicate the steps involved in the main pathways and dashed lines indicate alternative signaling steps. Created with BioRender.com.

Among all the ways in which necroptosis can be triggered, the TNF-induced pathway is the best characterized (Figure 1). Here, the activation of TNF Receptor 1 (TNFR1) is followed by the formation of complex I, a membrane-associated signaling platform composed of adaptor proteins, including the Receptor Interacting Protein Kinase 1 (RIPK1), Tumor necrosis factor Receptor type 1-Associated Death Domain (TRADD), the E3 ligases cIAP1 and 2, and Linear Ubiquitin chain Assembly Complex (LUBAC) [17]. The assembly of this complex is regulated by different polyubiquitin chains that allow the recruitment and activation of the Nuclear Factor ‘Kappa-light-chain-enhancer’ of activated B-cells (NF-kB) and Mitogen-Activated Protein Kinases (MAPKs) for subsequent pro-survival and inflammatory signaling [18].

Under the conditions in which RIPK1 is deubiquitinated, the alternative complex IIb (also known as the ripoptosome) is then formed [19]. Complex IIb consists of caspase-8, RIPK1, Fas-Associated via Death Domain (FADD), and different isoforms of cellular FADD-like IL-1β-converting enzyme Inhibitory Protein (cFLIP) [15]. Caspase-8 connects complex IIa to intrinsic apoptosis, as it mediates the cleavage of downstream caspases and the BH3-Interacting Domain (BID) protein [20,21]. On the other hand, TNF has also been shown to induce the cleavage of Gasdermin D (GSDMD) downstream of caspase-8 activation, leading to pyroptosis [22]. Caspase-8 normally restricts the activation of RIPK1 [23] and RIPK3 [24]. However, under conditions of caspase-8 inhibition (either pharmacologically or by the action of specific pathogens) [25,26], RIPK1 is recruited to form a third complex called the necrosome. The canonical necrosome is composed of RIPK1 and RIPK3 bound together by their RIP Homotypic Interaction Motif (RHIM) domains and assembled into a fibril-like structure [27]. Downstream of RIPK1 phosphorylation, RIPK3 is activated by autophosphorylation and then phosphorylates MLKL. This activation step induces the conformational change required for the assembly and translocation of MLKL oligomers to the membrane. Once there, MLKL oligomers mediate plasma membrane permeabilization by a mechanism that is not fully understood, leading to cell lysis as a result of the osmotic imbalance [28]. 

### 2.2. TRAIL-, Fas/CD95-, and IFN-Dependent Manners to Induce Necroptosis

The ripoptosome–necrosome axis is also effective in TNF-Related Apoptosis-Inducing Ligand (TRAIL) and Fas ligand pathway stimulation, both converging in RIPK1 activation [29,30,31] (Figure 1). In both cases, after ligand binding, the receptor trimerizes at the plasma membrane, and the Death-Inducing Signaling Complex (DISC) assembles. The DISC’s core is mainly composed of FADD and caspase-8 and can include other associated molecules, such as TRADD and RIPK1 [32]. Once the DISC is formed, RIPK1 is recruited to this complex and activated. Then, if caspase-8 is active, the subsequent steps for apoptosis take place. Otherwise, RIPK1 would form the necrosome with RIPK3, and necroptosis could occur [33]. Likewise, the incorporation of RIPK1 into the necrosome is promoted by interferon (IFN) through the Janus Kinase/Signal Transducer and Activator of Transcription (JAK/STAT) signaling pathway. In this case, the JAK/STAT pathway induces the transcription of the gene coding for the RNA-activated Protein Kinase (PKR), which interacts with RIPK1 to enable necrosome formation [34]. 

### 2.3. The Non-Canonical Necrosome

RIPK3 and MLKL can also be activated by RHIM domain-containing proteins other than RIPK1, constituting what is known as the non-canonical necrosome (Figure 1). One of these RHIM domain-containing proteins is Zipcode Binding Protein 1 (ZBP1), which binds to Z-DNA and Z-RNA nucleic acids [35,36,37]. Similarly, TIR-domain-containing adapter-inducing interferon-β (TRIF), which is activated upon the stimulation of Toll-Like Receptors 3 and 4 (TLR3 and TLR4), can induce the assembly of the necrosome [12]. In both cases, the inhibition of caspases is key for ZBP1 and TRIF-mediated necroptosis; otherwise, alternative cell death pathways can take over. For instance, LPS stimulation can trigger TLR4 signaling and activate the NLR family Pyrin domain containing 3 (NLRP3) inflammasomes and caspase-1 for the subsequent GSDMD cleavage and the induction of pyroptosis [22]. Intrinsic apoptosis can occur in this context as well, as TLR signaling can induce the activation of caspase-8 [38]. Furthermore, ZBP1 has also been implicated in LPS-induced cell death and can promote caspase-8 and inflammasome activation via its RHIM–RHIM domain interaction with RIPK1 [39]. Upon TLR stimulation, caspase-8, RIPK3, and MLKL are possible regulators of the NLRP3 inflammasome [40,41,42]. Thus, apoptosis, necroptosis, and pyroptosis are three types of intertwined regulated cell death that share common triggers and converge in the formation of protein platforms that serve as a decision point to determine the fate of the cell. 

## 3. Structural Requirements for MLKL Necroptotic Function

**Figure 2:**
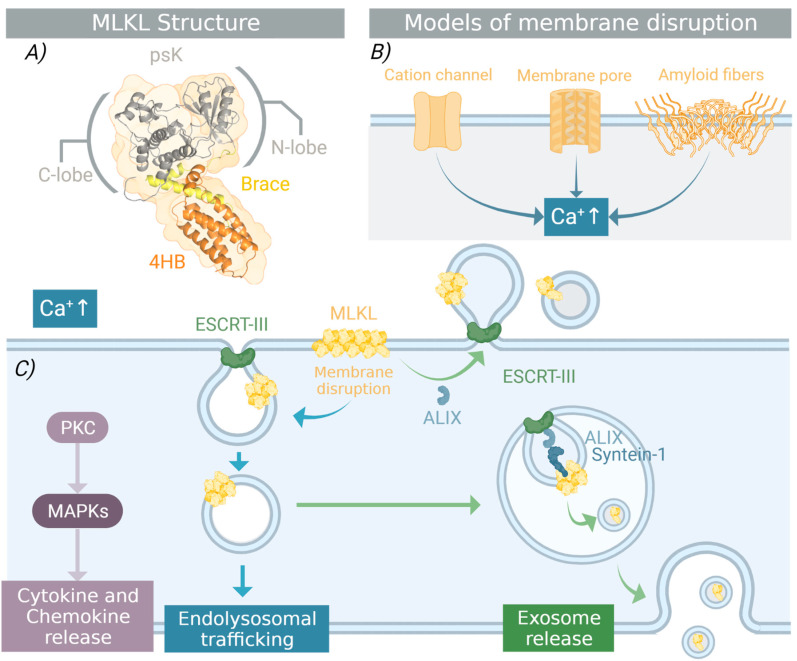
Structure and function of MLKL. (**A**) The MLKL protein structure (PDB: 4BTF). (**B**) The currently proposed models for plasma membrane permeabilization by MLKL include cation channel activation, pore formation, and disruption by amyloid fiber accumulation. (**C**) All of them converge in ion flux alterations and the increase in intracellular Ca^2+^ levels. Intracellular Ca^2+^ acts as a messenger that connects MLKL activity with other processes, such as membrane repair machinery (e.g., ESCRT-III and ALIX-Syntein-1 systems), the activation of PKC and subsequent signaling through the MAPKs, as well as cell death-independent processes, such as cytokine and chemokine production. Membrane repair is central to counterbalancing the accumulation of the activated MLKL in the cell. Created with BioRender.com.

MLKL structure is composed of a four-helix bundle (4HB) and a pseudokinase (psK) domain, linked by an interdomain brace region [43] (Figure 2A). The 4HB is known to be responsible for the killing function of the protein, while the psK domain has a regulatory role. Together with the brace interdomain, the psK is important for transmitting the structural rearrangement that occurs upon phosphorylation [43]. After MLKL is recruited to the necrosome and activated by RIP3K-dependent phosphorylation, it undergoes a conformational change that reorients the 4HB domain and the brace region. This allows the oligomerization of the protein, followed by the permeabilization of the plasma membrane and subsequent cell lysis. In the following section, we will summarize what is known about the molecular events taking place during necroptosis regarding MLKL activation, stressing the key aspects that remain a matter of debate in the field.

### 3.1. MLKL and RIPK3: Till Death Do Them Part

The nature of the interaction between MLKL and RIPK3 and their dynamics before and during necroptosis are still controversial. Structural studies of the human and mouse MLKL indicate that there may be significant differences between these orthologs because both proteins are not interchangeable [44]. It has been proposed that, in humans, MLKL and RIPK3 reside in a stable complex in the cytoplasm via their C-lobe interactions [45]. In agreement with this view, the basal interaction of RIPK3 and MLKL in humans is stable enough to co-immunoprecipitate. During necroptosis, these RIPK3:MLKL complexes are recruited to form the necrosome, appearing as cytoplasmic clusters, where MLKL undergoes a conformational change followed by its disengagement from RIPK3 [46]. In mice, however, the interaction between MLKL and RIPK3 seems to be less stable, resulting in a subtle relocation of MLKL into cytoplasmic clusters during necroptosis observed by microscopy [47]. As a consequence, the isolation of mouse RIPK3:MLKL complexes remains an experimental challenge under both basal and necroptotic conditions. These differences in the RIPK3–MLKL interaction between mice and humans might explain the structural discrepancies regarding MLKL activation in both species.

### 3.2. Post-Translational Modifications Governing MLKL Function

RIPK3-mediated phosphorylation of specific residues is a critical step in the activation of MLKL. Essential residues include T357/S358 in human MLKL [48] and S345 in mouse MLKL [2]. Besides them, additional RIPK3-dependent phosphorylation sites have been identified in MLKL. For instance, in mouse MLKL, the S347 in the activation loop is known to have an accessory role [2]. Moreover, it has been proposed that the phosphorylation of S228 and S248 in the N-lobe of human MLKL negatively regulates necroptosis [45]. Additionally, different RIPK3-independent phosphorylation sites of MLKL have been reported. However, many of them have not been characterized in terms of their function, and the kinases involved have not been identified. Of note, the TYRO3, AXL, and MERTK (TAM) kinases have been proposed to mediate the phosphorylation of Y376 in MLKL, a step that appears essential for its oligomerization and necroptosis [49].

Besides phosphorylation, the role of MLKL ubiquitylation (or ubiquitinylation) has recently emerged as another type of post-translational modification (PTM) required for its regulation. Ubiquitylated variants of MLKL have been specifically detected after necroptosis stimulation in different studies [50,51,52]. Although there is a clear correlation between the phosphorylation and activation of MLKL and the appearance of a band pattern as an indicator of ubiquitylation, the nature and function of this PTM remain unknown. One study suggested that specific ubiquitylation of K219 is important for the activation of MLKL and sensitizes cells to necroptosis [50]. In contrast, another study contemplates the possibility of the role of MLKL ubiquitylation as a mechanism to induce its downregulation after activation by promoting its degradation [51]. A third study focused on the biological role of this PTM and proposed that it might have a regulatory function to mediate the killing of intracellular bacteria during infection [52]. This topic is currently a subject of intense investigation and is further reviewed elsewhere [7,53].

### 3.3. Oligomerization and Membrane Translocation

Upon necroptosis, phosphorylated MLKL forms oligomers that translocate to cellular membranes. However, the mechanism and structural requirements of this process are still under debate. Although the exact stoichiometry of MLKL oligomers is not well-established, different studies suggest that they can range from trimers, tetramers, hexamers, and octamers, to higher-order oligomers [54]. Furthermore, it is still unclear which structural elements of MLKL contribute to or directly participate in different oligomerization interfaces. In this regard, a critical role of the interdomain brace in the formation of homotrimers in human MLKL has been suggested [43]. The authors propose a model in which the two-helix interdomain is key for communicating the structural change that first occurs in the psK domain upon RIPK3-dependent phosphorylation, which results in the release of the 4HB killing domain. Within this brace region, the residues T165 and L166 were shown to be especially important for oligomer assembly and necroptosis [43]. Another study extended this model and proposed that human MLKL assembles as a tetramer in a configuration in which not only the brace region but also the 4HB and the C-lobe of the psK domain participate in the oligomerization interface [43]. However, the specific amino acid residues participating in MLKL protomer–protomer interactions still need to be unveiled.

The molecular principles that determine MLKL translocation to cellular membranes also remain unclear. Early studies employing cellular fractionation identified the plasma membrane as the main target of MLKL activity [55]. This picture has been recently challenged with the identification of the intracellular vesicle trafficking system and microtubule-actin-Golgi machinery as sites for MLKL transport in the cell [6,56]. Whether these cellular compartments participate in MLKL movement to or from the plasma membrane is still puzzling. A study based on super-resolution microscopy experiments showed that phosphorylated MLKL migrates along with tight junction proteins toward the periphery of the cell and accumulates at the plasma membrane [6]. 

## 4. The Necroptotic and Non-Necroptotic Functions of MLKL

In the last decade, our knowledge about how MLKL is regulated has dramatically increased. The clear definition of the hallmarks and structural determinants required for its activation have opened new avenues on our journey to unveil the job of MLKL in the cell. Still, how MLKL promotes the final and key step of plasma membrane permeabilization during necroptosis and how its activity is related to its possible non-deadly roles remain highly controversial. Next, we will discuss current models that explain the biological function of MLKL.

### 4.1. Punching Holes in the Plasma Membrane

There is a consensus that the plasma membrane translocation of MLKL oligomers is mediated by the interaction between the phosphatidylinositol phospholipids and a cluster of positively charged residues in the 4HB domain [57]. This notion is also supported by a recent study showing that binding of a specific monobody to the α4-helix of the 4HB domain blocks the membrane translocation of human MLKL oligomers [58]. Specifically, residues D107, E111, and L114 were identified to be at the binding site of the monobody to MLKL, and their mutation impaired the membrane translocation. Interestingly, this study contemplated the possibility of the existence of other interaction partners or receptors, rather than lipids, of MLKL in the plasma membrane. This hypothesis is supported by the evidence that D107 and E111 do not seem to be involved in the interaction of MLKL with lipids [58]. In any of these scenarios, the binding of MLKL to membranes is thought to promote the acquisition of the active conformation by promoting the reorientation of the brace region [59]. 

Different models of how MLKL mediates plasma membrane permeabilization have been suggested over the years (Figure 2B). One model proposed that MLKL forms ion channels. More specifically, one study suggested that MLKL forms cation channels that are mostly permeable to Mg^2+^ [60], while others reported the possibility of MLKL activating endogenous Ca^2+^ channels [55]. Alternatively, MLKL could directly mediate necroptosis by forming membrane pores [61]. Here, MLKL would either act as a surfactant on the membrane or form stable pores after its association and insertion into the lipid bilayer [62]. Another possibility is that the MLKL forms amyloid-like fibers stabilized by disulfide bonds. These structures could directly affect the integrity of the membrane or function as platforms for the recruitment of other auxiliary proteins [63].

Despite these remaining discrepancies, most of these models agree on the role of MLKL as the direct mediator of membrane permeabilization in necroptosis. Furthermore, it is clear that the alterations of ion fluxes across the plasma membrane represent a downstream consequence of MLKL activity. Among these ions, Ca^2+^ is of special interest due to its versatile role as a second messenger in the cell (Figure 2C). A rise in the cytosolic Ca^2+^ during necroptosis has been linked with the activation of the ESCRT-III machinery [64] and the ALIX-syntenin-1-mediated exocytosis system [65] that compete against MLKL-induced membrane damage to prevent cell death. More recently, it has been proposed that, under sub-lethal conditions, the Ca^2+^ influx at the damage sites of necroptotic cells can mediate the activation of Protein Kinase C (PKC), resulting in the initiation of a complex signal transduction network involving NF-kB and MAPK signaling [66]. Ca^2+^-dependent signaling processes that are activated downstream of MLKL-induced membrane damage can play an active role in the regulation, not only of cell death but also in the production of chemokines and cytokines. In this context, MLKL function could be understood as a complex process beyond cell death. What is becoming clearer is that sub-lethal MLKL can potentially trigger other cellular types of machinery, contributing to different defense mechanisms that allow individual cells and tissues to cope with damage.

### 4.2. One Protein with Many Faces

Aside from its function as an executor of necroptosis, MLKL has been associated with other cellular processes independently of cell death. Still, most of these alternative roles of MLKL are also linked to the ability of this protein to disturb cellular membranes under sub-lethal conditions. For instance, it has been shown that MLKL contributes to extracellular vesicle generation and that this function is independent of RIPK1 and RIPK3 activation [56,67]. In this scenario, it can be envisioned that MLKL accumulation in the plasma membrane leads to its incorporation in the extracellular vesicles as a mechanism to antagonize its necroptotic activity. Related to its localization in extracellular vesicles, MLKL has been implicated in the clearance of intracellular bacteria independently of necroptosis [52,68]. Targeting MLKL to endosomes seems to promote intracellular trafficking of bacteria, such as *Listeria monocytogenes* and *Yersinia enterocolitica*, to lysosomes to control infection [52]. Interestingly, in a yeast model, the expression of the human MLKL disrupts autophagy and endosomal trafficking by interfering with the mammalian target of the rapamycin complex 1 (TORC1) signaling pathway [69].

MLKL seems to also be implicated in the induction of membrane remodeling. Related to its capability to target the plasma membrane, MLKL plays an essential role in the induction of phosphatidylserine (PS) exposure to the cell surface, observed upon the activation of necroptosis [64]. In support of this, various studies have shown that the silencing of MLKL inhibits PS exposure in necroptosis [70,71]. This change in membrane asymmetry is a common process that promotes the recognition of dying cells by macrophages and, thus, the activation of the immune system. It is uncertain whether MLKL directly mediates this process or functions as a regulator of cellular scramblases. For instance, the induction of scramblase activity could be linked to MLKL activity and the early increase in cytosolic Ca^2+^ observed in necroptotic cells [54]. 

Furthermore, it has been demonstrated that MLKL participates in response to nerve injury by promoting the demyelination process that allows nerve regeneration [72,73]. Authors show how, in the absence of cell death, sciatic nerve injury induces MLKL expression in Schwann cells, the RIPK3-independent phosphorylation of S441, and the translocation of MLKL to myelin sheath membranes. Once there, MLKL is proposed to mediate myelin breakdown, a process that is required for subsequent axonal regeneration. Interestingly, the sphingolipid sulfatide, rather than phosphatidylinositol phospholipids, has emerged as the putative lipid receptor of MLKL in these membranes. In this context, the lipid specificity of MLKL would be modulated by phosphorylation at different sites and determine its role as a perturbator of membranes in different biological settings. 

The induction of necroptosis also promotes MLKL translocation to the nucleus. This process seems to be unrelated to its function as a death executor, as it precedes plasma membrane disruption and necroptosis. Nevertheless, translocation to the nucleus relies on MLKL activation and phosphorylation, which likely leads to the exposure of a nuclear localization site located in the 4HB domain [74] and plays a role in necroptosis regulation [75]. Although it is not clear whether this function is related to cell death, the fact that MLKL harboring mutations umpiring nuclear translocation is still capable of killing the cell suggests that nuclear translocation may be dispensable for necroptosis. Further evidence supports that MLKL could act as a transcriptional regulator due to its ability to translocate into the nucleus and interact with nuclear proteins. One identified partner of MLKL in the nucleus is the RNA-Binding Motif Protein 6 (RBM6). It has been proposed that the interaction of MLKL with RBM6 promotes the mRNA stability of adhesion molecules in endothelial cells insensitive to necroptosis [76]. 

## 5. MLKL Is a Potential Target for Treating Human Diseases

Evidence obtained in animal models has linked necroptosis mediators with the pathophysiology of various diseases involving tissue damage and inflammation [77,78]. However, the specific role of necroptosis and MLKL in humans remains elusive. In the following section, we will discuss evidence supporting the involvement of MLKL in the pathology of several human diseases. 

An in silico analysis of MLKL expression in publicly available databases revealed marked differences in *MLKL* mRNA levels in different tissues upon various stimuli [79]. In this regard, it has been shown that MLKL is transcriptionally upregulated by interferons in different types of cells, such as cancer cells [80], lung epithelial cells [81], and bone-marrow-derived macrophages [82] in response to inflammatory stimuli. This evidence supports the notion that cellular environmental cues, such as infection, tissue injury, inflammation, and cancer, result in a higher expression of MLKL. On the other hand, *RIPK3* gene expression tends to remain at a comparable level in inflammatory contexts. As MLKL can be expressed in the absence of RIPK3 in many cell lines and tissues, it could potentially be linked to functions that are independent of its known upstream kinase or necroptosis [79]. This fascinating alternative would broaden the relevance of MLKL to cellular function and warrants further investigation.

**Figure 3:**
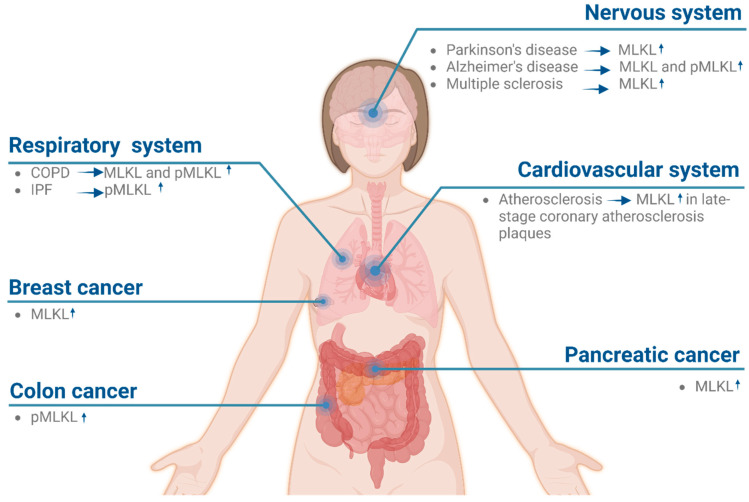
Evidence linking the involvement of MLKL upregulation with human diseases. MLKL has a crucial role in the pathogenesis of several distinct diseases, including conditions in the nervous, respiratory, and cardiovascular systems. Upregulation or activation of MLKL was also shown to have a role in different types of cancers, such as breast cancer, colon cancer, and pancreatic cancer. Arrows indicate an increase (↑) in MLKL levels. pMLKL: phosphorylated MLKL. Created with BioRender.com.

The analysis of human samples using antibodies that recognize either MLKL or its activated phosphorylated version raises the possibility of its involvement in different pathologies. These include neurodegenerative, cardiovascular, and pulmonary diseases (Figure 3). Supporting its role in the diseases of the nervous system, both MLKL and phosphorylated MLKL levels were found to be elevated in derived *postmortem* tissues of patients with Parkinson’s disease and samples from the brains of patients with Alzheimer’s disease [83,84,85]. Moreover, activated MLKL has been detected in pathological samples from the cortical lesions of patients with multiple sclerosis [86]. Experiments in mice also support the role of MLKL during brain damage. For example, it has been shown that defective necroptosis resulting from the knockout of MLKL promotes the switch from a macrophage M1-like neurotoxic state to M2-like neuroprotective state during ischemic stroke. In the context of cardiovascular diseases, activated MLKL was specifically detected in late-stage coronary atherosclerosis plaques [87]. In line with this notion, experiments with a prothrombotic mouse model have shown that there is a significant reduction in lesion size in the cortex when MLKL is knocked out [88]. Finally, evidence also points to a role for MLKL in Coronary Obstructive Pulmonary Disease (COPD) and Idiopathic Pulmonary Fibrosis (IPF), as active MLKL is elevated in the lung tissues of the patroness of these diseases [89,90]. Therefore, targeting MLKL can potentially attenuate airway inflammation, emphysema, and airway remodeling. 

The role of MLKL in cancer is still puzzling, as necroptosis could have both antitumorigenic and pro-tumorigenic roles. In fact, the expression of necroptosis mediators can be either downregulated or upregulated in different types of cancer cells. For instance, studies on patients with pancreatic cancer [79] found that in addition to RIPK1/3, MLKL protein expression was also elevated [91]. In line with this, increased levels of MLKL were detected in the invasive form of human pancreatic cancer tissue [92]. In contrast, some studies associate a low level of MLKL expression with a reduction in the overall survival of numerous cancer-resected patients, including those suffering from pancreatic adenocarcinoma [93], cervical cancer, and breast cancer [94]. Moreover, it has been shown that activated MLKL is elevated in colon cancer patients and the tumor tissues of the esophagus and neck squamous cell carcinoma [95]. Furthermore, some studies associate the downregulation of the mRNA levels of *MLKL* with the propagation of several types of acute myeloid leukemia [96]. Lastly, the downregulation of *MLKL* transcript has been linked with poor prognosis in breast, colorectal, and cervical cancers [79,94,97,98].

## 6. Inhibition of MLKL with Small Molecules

Identifying and discovering MLKL as a critical factor in disease has opened up new research and drug discovery avenues. Due to its unique and central role in necroptosis, MLKL quickly emerged as an attractive molecule to specifically targets this pathway. Inhibiting necroptosis by targeting MLKL can potentially present several advantages. First, it is a pseudokinase with pore-forming activity, and therefore, its function differs from the widespread kinase activity of other pathway components, such as RIPK1/3. Modulation of MLKL could be achieved not only by targeting the pseudokinase domain, which shares some structural similarities with canonical kinases, but also by other regions that are directly involved in their activity (i.e., the 4HB or the brace). Second, it is the most downstream effector in necroptosis, and, in contrast with upstream mediators, it is not connected to other cellular pathways. In this regard, targeting MLKL can potentially circumvent the issues observed with the inhibition of RIPK1 and RIPK3. For instance, the inhibition of RIPK1 can also affect apoptosis, while the inhibition of RIPK3 results in toxicity due to the induction of aberrant apoptosis.

Numerous studies have emerged that identify and develop new MLKL inhibitors, including small molecules targeting different protein sites. Small molecules that bind to or inhibit MLKL can be classified into three major categories: (1) inhibitors that bind in an irreversible mode to the 4HB domain, (2) inhibitors that interact with the ATP-binding site located in the psK, and (3) allosteric and reversible binders that target the killer 4HB domain (Table 1). However, they still suffer from several limitations, including moderate potency, narrow structure–activity relationship (SAR), and undesirable off-target effects. Therefore, current molecular modulators of MLKL are limited to early drug development stages and are mainly applied to research purposes. Next, we will summarize the main properties of the MLKL inhibitors that have been discovered or are under development.

### 6.1. Irreversible Inhibitors

Necrosulfonamide (NSA) is a small molecule that was identified as a potent necroptosis inhibitor in a cell-based high-throughput screen [99]. Thanks to this study, MLKL, which emerged as the molecular target of NSA, was recognized as the main effector of necroptosis. Since then, NSA has been widely used in experimental research as a tool to study necroptosis in cells. In a second study from the same screen, another compound was selected as a potent necroptosis inhibitor and further optimized into a series of new MLKL inhibitors. From this study, a new compound named TC13172 emerged as a potent MLKL inhibitor with a nanomolar potency (Table 1) [100]. Both NSA and TC13172 inhibit necroptosis in human cell lines, however, not in mouse cells. The high species selectivity of these inhibitors is due to their mechanisms of action, as they specifically target C86 of MLKL, located in the α4-helix of the 4HB domain and are unique to the human orthologue. Therefore, these inhibitors are not suitable for basic research or pre-clinical studies employing mice or murine cells due to the lack of humanized mouse models. As these are essential steps in drug development, this type of inhibitor has limited perspectives in their use for the treatment of human diseases. More recently, a reconstitution model of the human necrosome in yeast has been developed as an alternative way to study the effect of small molecule inhibitors, such as TC13172, which resulted in a decreased cell death in yeast after necroptosis induction [108]. 

Despite their high potency, NSA and TC13172 suffer from different drawbacks. In addition to its narrow SAR, NSA shows a moderated selectivity derived from binding to a surface cysteine. NSA also exhibits cross-reactivity with other proteins, including mouse and human GSDMD, during pyroptosis [101] and the trafficking of the tight junction protein, ZO-1, during TNF-induced necroptosis [6]. TC13172 can react readily with nucleophilic reagents due to the presence of a methylsulfonyl group, which potentially facilitates off-target activity and poor metabolic stability in vivo. In more recent attempts to improve covalent MLKL inhibitors, a new family derived from TC13172 was generated and characterized. In this new generation of inhibitors, the xanthine core of TC13172 was replaced with an uracil core [102], resulting in better drug-like properties, such as higher stability and lower potential off-target effects, albeit with lower potency (Table 1). 

Although NSA, TC13172, and uracil derivatives all bind to C86 of human MLKL, they seem to follow different mechanisms of inhibition. Apart from binding C86, NSA forms a π-cation interaction with L157 located in the second brace helix. On the other hand, TC13172 acts by stabilizing the packing of α-helix 6 from the brace region against the 4HB through the formation of π–π-stacking interactions with F148 [103]. In the case of the uracil-derivative compound **56**, covalent binding to C86 occurs via a replacement of the 6-CL group, which leads to the establishment of π–π interactions between its core and F148 of MLKL [102].

Despite the covalently binding MLKL through similar mechanisms, NSA, TC13172, and uracil-derivative compounds affect the hallmarks of MLKL activation slightly differently. None of them affect the activation of the upstream kinases RIPK1/3 or affect MLKL phosphorylation mediated by RIPK3. In fact, NSA causes the partial inhibition of MLKL oligomerization, an effect that is stronger than the one observed for the uracil derivatives. On the other hand, TC13172 completely blocks the oligomerization of MLKL. Of note, all the inhibitors of this class completely block translocation to membranes [102].

### 6.2. Inhibitors That Target the psK Domain

Given the central role of the psK domain in the regulation of MLKL activation, targeting this site was an early strategy for the development of necroptosis inhibitors. GW806742X, also known as compound **1**, was the first MLKL inhibitor of this class and was identified after screening against the psK domain of recombinant mouse MLKL (Table 1) [104]. In another complementary study, compound **2** was discovered as a more potent derivative of compound **1** that can bind to either the psK domain or the full-length human and mouse MLKL [105]. Mechanistically, compound **1** competes with ATP or ADP for binding to MLKL, further validated by its lack of interaction with MLKL K219M, which is a mutant with a defective ATP binding site. Compound **2** binds to the psK domain, specifically to the nucleotide-binding pocket, where it forms hydrogen bonds with different MLKL residues, such as E250, G349, and C286. Both compounds **1** and **2** inhibit necroptosis with very high potency, showing IC50 values in the nanomolar range and binding affinities in the low micromolar range [105]. However, despite these inhibitory profiles, this approach to developing MLKL inhibitors suffers from low specificity as a drawback because the ATP-binding site of MLKL shares structural similarities with other kinases and pseudokinases. In fact, compound **1** was initially described as an inhibitor for the protein kinase Vascular endothelial growth factor receptor-2 (VEGFR2) [106], and both compounds also bind to RIPK1 and RIPK3 with higher affinity than to MLKL. Therefore, their inhibitory effects do not rely only on their ability to target MLKL. In any case, these compounds represent potent inhibitors of necroptosis, with proven efficacy in the in vivo mouse model of TNF-induced Systemic Inflammatory Response Syndrome (SIRS) [105].

### 6.3. Fragment-Based Allosteric and Reversible Binders

The reversible and allosteric modes of interaction with the 4HB domain of MLKL characterize the third and last class of inhibitors under development (Table 1). This type of compound emerged after screening a fragment library against the recombinant 4HB domain of the human MLKL [27]. Compound **1**, with an indole moiety, was selected as a starting molecule that underwent further optimization. Improved derivatives (compounds **5** and **7**) were characterized by an enhanced binding affinity that was still in the micromolar concentration range (>50 µM). It was discovered that both compounds bind to a hydrophobic pocket that is located at the end of the 4HB domain, opposite to C86, the target residue of irreversible inhibitors of MLKL. Interestingly, the detergent nonyl-maltoside, which, together with phytic acid, acts as an activator of the 4HB domain and can compete with compound **5** for binding to MLKL. This evidence opened the possibility that these compounds could inhibit MLKL in cell-based assays. This notion is based on the similarity of these detergents with inositol phosphates, which are required for MLKL oligomerization and translocation to membranes [107]. Unfortunately, the activity of compound **5** could not be tested in cells due to its poor membrane permeability, and it was not active in an in vitro liposome leakage assay. Therefore, additional optimization is still required to prove their efficacy as MLKL inhibitors. 

## 7. Conclusions and Future Perspectives

MLKL is the most important player in the execution of necroptosis, a type of RN that is triggered under conditions in which the activity of caspase-8 is compromised. In recent years, the cell death field has progressed in understanding how MLKL is regulated to execute necroptosis. Answers to many open questions remain elusive, thus limiting our ability to modulate necroptosis in a targeted fashion to promote human health. How does interconnectivity with other forms of regulated cell death contribute to the outcome of MLKL activity in different physiological settings? What are the common nodes that determine cell fate or the decision of which type of cell death dominates? At the molecular level, we still do not know all the key cellular players that modulate MLKL function in different contexts or the exact mechanism of how this intriguing protein mediates membrane permeabilization and, ultimately, cell death. Can MLKL-mediated membrane perturbation lead to other cellular processes that are independent of its role as a killer? Furthermore, we are just starting to grasp the involvement of MLKL in different human pathologies. Finding out whether the deregulation of MLKL is relevant to the development of human diseases is essential to identifying which interventions could benefit from targeting MLKL and necroptosis. Ongoing efforts to elucidate the structural mechanisms of MLKL activation will enable future strategies to inhibit necroptosis for the treatment of life-threatening diseases.

## Figures and Tables

**Table 1:** Best characterized MLKL inhibitors and binders to date.

Class	Mechanism	Inhibitor	Structure	Potency	Effect on Hallmarks of MLKL Activation	Limitations	Ref.
Irreversible inhibitors	Covalent binding to the 4HB via C86	NSA	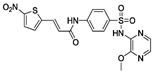	IC50 = 1.4 nM	- No effect on MLKL phosphorylation - Partial inhibition of MLKL oligomerization - Block MLKL membrane translocation	- Exclusive recognition of human MLKL - Off-target effects: they can bind to any reactive Cys in the cell (e.g., NSA binds to GSDMD, the executor of pyroptosis)	[99,100,101,102,103]
		Xanthine-derivatives ex:TC13172	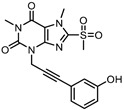	IC50 = 2 nM	- No effect on MLKL phosphorylation - Block MLKL oligomerization - Block MLKL membrane translocation		
		Uracil-derivatives	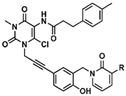 Cpd 56 R = H Cpd 66 R = OCH3	Cpd 56 IC50 = 82 nM Cpd 66 IC50 = 31 nM	- No effect on MLKL phosphorylation - Weak effect on MLKL oligomerization - Significant reduction of MLKL membrane translocation		
Targeting the ATP binding site	Non-covalent binding to the psK domain	Compound **1** or GW806742X	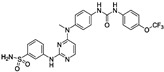	IC50 < 50 nM Kd = 0.53 µM	- Potential effect on MLKL phosphorylation due to targeting of RIPK1 and RIPK3	- Off-target effects: poor kinome selectivity. They also target RIPK1 and RIPK3	[104,105,106]
		Compound **2**	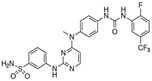	IC50 < 50 nM Kd = 1.8 µM			
Allosteric and reversible binders	Non-covalent binding to the 4HB domain	Compound **5**	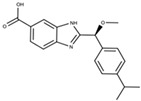	Kd = 50 µM	- Still not identified	- Low affinity (in the mM range) - Not tested in cells- No off-target effects tested	[107]

IC50: concentration required to inhibit 50% of cell death. Kd: Affinity constant of the interaction with recombinant MLKL.

## Data Availability

Not applicable.
